# Origins of Enhanced Enantioselectivity in the Pd-Catalyzed Decarboxylative Allylic Alkylation of *N*-Benzoyl Lactams ^†^

**DOI:** 10.3390/catal13091258

**Published:** 2023-08-30

**Authors:** Alexander Q. Cusumano, Tianyi Zhang, William A. Goddard, Brian M. Stoltz

**Affiliations:** 1 The Warren and Katharine Schlinger Laboratory for Chemistry and Chemical Engineering, Division of Chemistry and Chemical Engineering, California Institute of Technology, Pasadena, CA 91125, USA; 2 Materials and Process Simulation Center, Beckman Institute, California Institute of Technology, Pasadena, CA 91125, USA

**Keywords:** C(sp^3^)–C(sp^3^) cross-coupling, asymmetric catalysis, computation, allylic alkylation

## Abstract

We explore the origins of the marked improvement in enantioselectivity in the inner-sphere (PHOX)Pd-catalyzed allylic alkylation of *N*-benzoyl lactam nucleophiles over their carbocyclic counterparts. We employ density functional theory calculations to aid in the interpretation of experimental results. Ultimately, we propose that the enhancement in enantioselectivity arises primarily from noncovalent interactions between the substrate and ligand rather than secondary substrate chelation, as previously hypothesized.

## 1. Introduction

**Figure 1.**
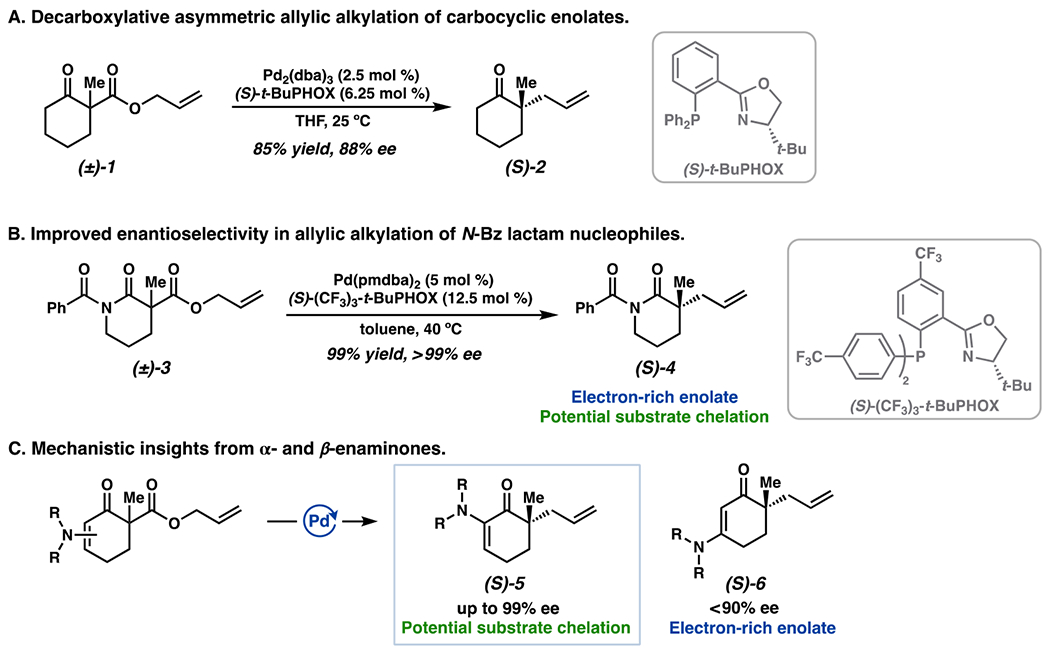
(**A**) The decarboxylative asymmetric allylic alkylation of cyclic ketone nucleophiles. (**B**) Allylic alkylation of *N*-benzoyl lactams. (**C**) Mechanistic insights from a- and b-enaminone substrate classes.

The Pd-catalyzed decarboxylative asymmetric allylic alkylation of *hard* enolate nucleophiles is a proven tactic for the formation of all-carbon quaternary stereogenic centers [1,2]. Employing chiral *tert*-butyl phosphinooxazoline (*t*-BuPHOX) ligands renders the transformation asymmetric, with an enantiodetermining inner-sphere reductive elimination [3–9] (Figure 1A). Despite extensive ligand optimization efforts, enantioenrichment of carbocyclic ketone products (**2**), derived from b-ketoesters (**1**) or enol carbonates, are generally limited to 80–90% ee. In contrast, *N*-benzoyl lactams (**3**) undergo the analogous transformation with markedly higher levels of enantioselectivity, often ≥99% ee (**4**) [10]. This represents a substantial increase in the effective difference in barrier height between diastereomeric enantiodetermining transition states (ΔΔG^‡^), from *ca*. 1.6 to >3.3 kcal/mol (Effective ΔΔG^‡^ calculated from Eyring equation at 40 °C. Note the effective ΔΔG^‡^ may not directly correspond to the free energy difference between only the two lowest diastereomeric transition states if multiple low energy conformeric transition states are present) (Figure 1B). Compared to their carbocyclic counterparts, the lactam substrate class would afford a potentially more electron-rich Pd-enolate, which may serve to reinforce a highly selective inner-sphere mechanism over a poorly selective outer-sphere process [7]. However, we also posited that the presence of an adjacent Lewis basic carbonyl group may enable additional interactions with the metal center. To independently examine each of these variables, we explored the a- and b-enaminone substrate classes (Figure 1C). Of note, a-enaminones (**5**) with a-heteroatom chelating groups retain the high levels of enantioselectivity of the *N*-benzoyl lactam substrate class [11], while b-enaminones (**6**) featuring more electron-rich enolates but lacking the ability to engage in hypothesized secondary interactions afford products in <90% ee [12]. These results suggest that the a-heteroatom-containing fragment of the substrate appears to play a key part in improving enantioselectivity. Here, we employ computational tools to elucidate this role, ultimately deepening our understanding of the origins of enantioselectivity in the inner-sphere allylic alkylation reaction.

## 2. Results and Discussion

**Figure 2.**
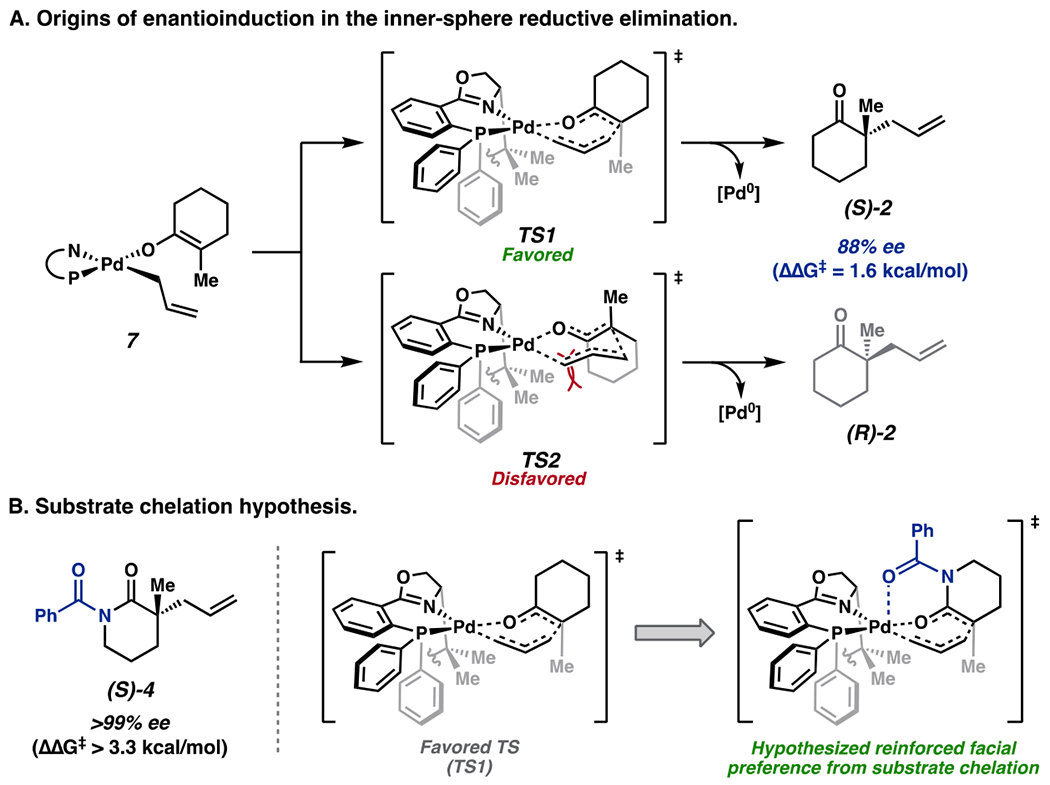
(**A**) Enantioinduction via inner-sphere reductive elimination. (**B**) Initial hypothesis for enhanced enantioenrichment of *N*-benzoyl lactam substrates as compared to cyclohexanones.

The enantiodetermining C–C bond formation in the (PHOX)Pd-catalyzed asymmetric allylic alkylation occurs via a seven-centered pericyclic transition state [7,13]. Enantioselectivity arises from preferential exposure of the *Re* face of the prochiral enolate ligand to the h^1^-llyl terminus (**TS1**) (Figure 2A). Bond formation from the *Si* face; (**TS2**) is disfavored due to steric incursions between the carbocyclic enolate backbone and the ligand scaffold. The dramatic improvement in enantioenrichment of *N*-benzoyl lactam products suggests an enhanced favorability of the analogous *Re* transition states over their *Si* counterparts. As similar levels of enantioselectivity were observed with a-enaminones (Figure 1C), we posited that such a-heteroatom-containing motifs may reinforce the *Re* facial preference through axial chelation with the Pd^II^ center in the reductive elimination transition state (Figure 2B, right). To further probe this hypothesis, we turned to computations.

**Figure 3.**
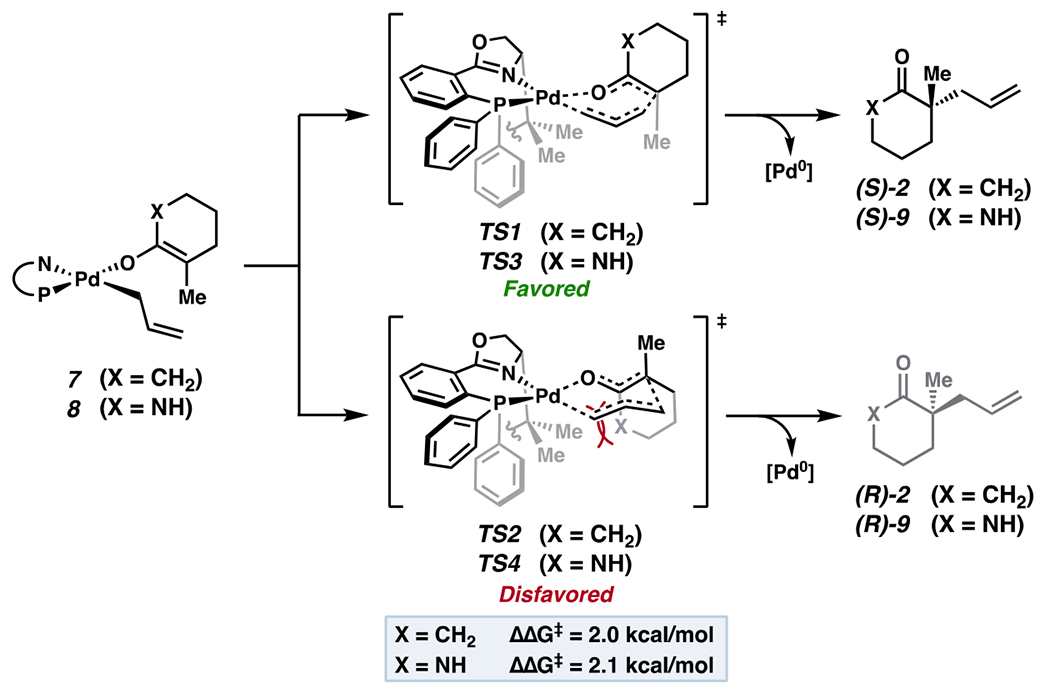
Minor effect of alteration in enolate electronics.

Beginning with enolate **7**, derived from carbocyclic substrate **1**, we find a 2.0 kcal/mol preference for **TS1** over **TS2** at the revDOD-PBEP86-NL/def2-TZVPP/SMD(PhMe)//r^2^SCAN-D4/def2-TZVP[Pd], def2-SVP level of theory—in accord with our prior studies (Figure 3) [7]. Maintaining a similar steric profile while perturbing enolate electronics with N–H lactam-derived enolate **8** did not significantly alter ΔΔG^‡^. Accounting for distribution across all conformers, enantiomeric excesses of 89% and 90% are computed, respectively (experimentally, the corresponding N–H lactam is not compatible in the transformation).

**Figure 4.**
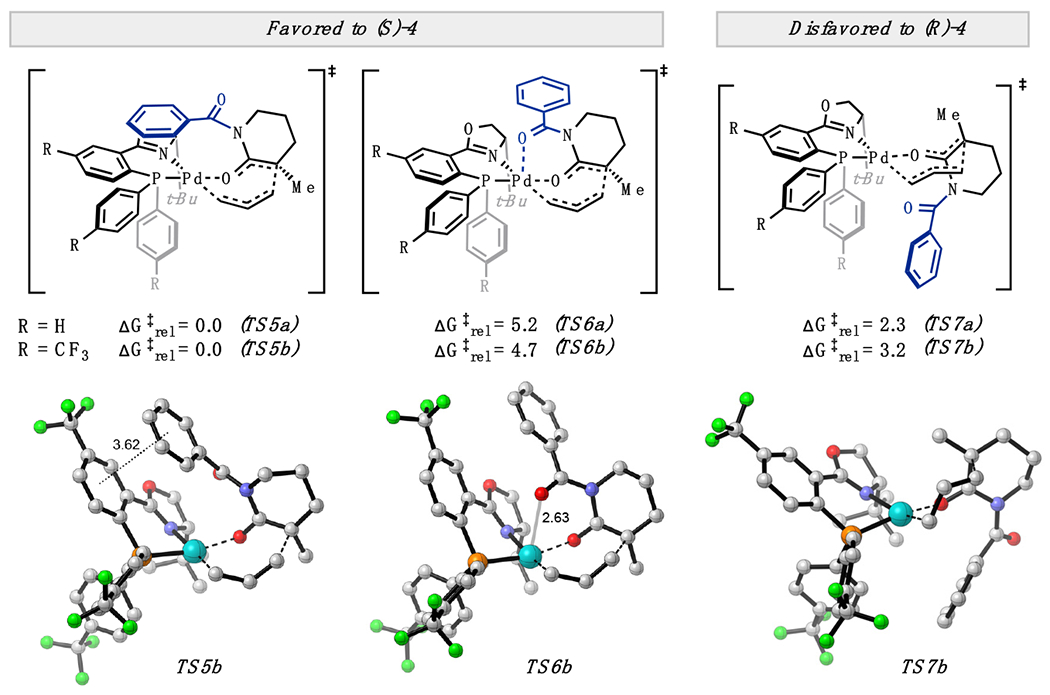
C–C bond-forming transition states for the *N*-benzoyl lactam substrates.

We then explored the effect of *N*-substitution on the relative free energies. *N*-benzoyl substitution affords two low-energy transition states from the favored *Si* face—one conformer with the flanking carbonyl of the benzoyl group oriented away from (**TS5**) and another toward (**TS6**) the metal center (Figure 4). **TS6** is reminiscent of our chelating heteroatom hypothesis (Figure 2B). However, **TS5** is computed to be favored over **TS6** by 5.2 and 4.7 kcal/mol with (*S*)-*t*-BuPHOX and (*S*)-(CF_3_)_3_-*t*-BuPHOX ligands, respectively. With regard to enantioselectivity, **TS5** is favored over the lowest energy *Si* face transition states (**TS7**) by 2.3 and 3.2 kcal/mol with the (*S*)-*t*-BuPHOX and (*S*)-(CF_3_)_3_-*t*-BuPHOX ligands, respectively. Computed enantiomeric excesses of 95% and 99% are found when accounting for all transition state conformers. The additional increase in ΔΔG^‡^ of 0.9 kcal/mol with incorporation of *p*-CF_3_ groups may arise from increasing favorable electrostatic interactions between the benzoyl and PHOX ligand arene quadrupoles in **TS5b**. We note the electron-poor (*S*)-(CF_3_)_3_-*t*-BuPHOX ligand is also crucial in promoting the inner-sphere mechanism discussed herein over less selective outer-sphere pathways.

While the computed trends in enantiomeric excess are in accord with experimental values, the energetic preference for **TS5** over **TS6** mandates a re-evaluation of our initial hypothesis regarding axial chelation. **TS6a** and **TS6b** feature axial Pd–O distances of 2.64 and 2.63 Å (compared to equatorial Pd–O distances of 2.20 and 2.19 Å), highlighting the lack of strong axial binding of the carbonyl oxygen. While the s-donating oxygen lone pair is repelled by the occupied axially-oriented 4d(z^2^) orbital of the d^8^ Pd^II^ center, mixing with the empty 5p(z) orbital may contribute to a partial s bonding interaction (for further discussion on such 3-center 4-electron bonding arrays in d^8^ complexes, see [14]). Geometric constraints inhibit π bonding interactions with the carbonyl group. We also suspect an electrostatic contribution to the weak Pd–O axial binding. However, four-coordinate **TS5a** and **TS5b** are the favored conformation of the *Si* transition states by a considerable margin.

**Figure 5.**
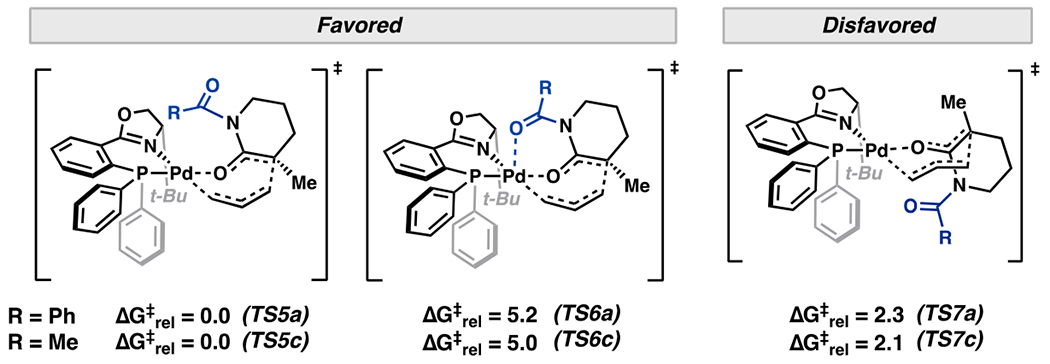
Comparison of relative barriers between diastereomeric C–C bond-forming transition states in both *N*-Bz and *N*-Ac lactams.

A similar trend is observed with *N*-acetyl lactam transition states (**TS5c** and **TS6c**), highlighting that the preference for four-coordinate transition states is not a conformational artifact of the benzoyl arene (Figure 5). Additionally, *N*-carbamate groups (Boc, CBz, and Fmoc) that are more Lewis basic lead to reduced enantioselectivities of 73–87% ee. Hence, axial chelation to the square planar Pd center in the reductive elimination does not appear to enhance enantioselectivity.

In lieu of secondary substrate chelation to Pd, we posit that the success of the benzoyl group lies in its ability to adopt a parallel orientation to the open face of the PHOX backbone in **TS5**. However, the *tert*-butyl group occupies this site in the *Si* transition state (**TS7**). Hence, this low-energy orientation is not accessible, and a large energetic penalty is incurred in C–C bond formation from the *Si* face. The results presented herein suggest that the improved enantioselectivity observed in the *N*-Bz class of substrates is noncovalent in nature. While no evidence of secondary substrate chelation is found for *N*-Bz lactams, such interactions may prevail in other substrate classes. The detailed investigation of these systems will be reported in due course.

## 3. Computational Details

All quantum mechanics calculations were carried out with the ORCA program (version 5) [15]. The r^2^SCAN functional [16] paired with D4 dispersion corrections [17], hence-forth referred to as r^2^SCAN-D4, was employed for geometry optimizations and harmonic frequency calculations. Similar geometries were obtained across a variety of density functionals. For geometry optimization and harmonic frequency calculations, Pd is described by the def2-TZVP basis set [18] and the ECP28MWB small-core (18 explicit valence electrons) quasi-relativistic pseudopotential [19], while C, H, and P are assigned the def2-SVP basis. Diffuse functions are added to O, N, and F (ma-def2-SVP). All Hessians were computed analytically. Stationary points are characterized by the correct number of imaginary vibrational modes (zero for minima and one for saddle points). Cartesian coordinates of all optimized structures are included as “.xyz” files and are available online in a compressed zip file format (see Supplementary Materials).

Electronic energies are further refined with single-point calculations employing the revDOD-PBEP86-NL double hybrid functional with non-local dispersion corrections [20] and the def2-TZVPP basis set on all atoms (with the ECP28MWB pseudopotential for Pd) with additional diffuse functions on O, N, and F (ma-def2-TZVPP). Solvation was accounted for with the SMD solvation model for toluene. Similar results were obtained from single-point calculations employing the range-separated hybrid wB97M-V functional [21]. To check for basis set superposition error, single-point calculations of select transition states (**TS5**, **TS6**, and **TS7**) were carried out with revDOD-PBEP86-NL and wB97M-V functionals paired with the quadruple-z quality (ma-)def2-QZVPP basis set. Similar results are obtained; hence, we recommend the more computationally tractable triple-z quality basis set for this application. Final Gibbs free energies were obtained by applying thermodynamic corrections obtained at the optimization level of theory to these refined electronic energies. Thermodynamic corrections from harmonic frequency calculations employ the quasi-rigid rotor harmonic oscillator approach to correct the breakdown of the harmonic oscillator approximation at low vibrational frequencies [22].

All stereochemical perturbations (*Re/Si*, chair/boat, axial/equatorial) and conformations (carbonyl distal, carbonyl proximal) are considered for each reaction pathway. Computed enantiomeric excess accounts for contributions from all considered transition states weighted by their final relative Gibbs free energies at 40 °C. All quantum mechanical data are included online in the supplementary Excel file (see Supplementary Materials).

## Supplementary Material

supplementary material

XML

## Data Availability

The authors confirm that the data supporting the findings of this study are available within the article and its Supplementary Materials.
